# Meso-Structuring of SiCN Ceramics by Polystyrene Templates

**DOI:** 10.3390/nano5020425

**Published:** 2015-03-27

**Authors:** Julia-Katharina Ewert, Christine Denner, Martin Friedrich, Günter Motz, Rhett Kempe

**Affiliations:** 1 Anorganische Chemie II (Catalyst Design), Universität Bayreuth, 95440 Bayreuth, Germany; E-Mails: julia.ewert@uni-bayreuth.de (J.-K.E.); christine.denner@uni-bayreuth.de (C.D.); martin.friedrich@uni-bayreuth.de (M.F.); 2 Institute of Ceramic Materials Engineering, Universität Bayreuth, 95440 Bayreuth, Germany

**Keywords:** SiCN, meso-structured, self-sacrifical template method, polystyrene particles

## Abstract

A simple one-pot synthesis of well-defined PS-silazane nano-composites (polystyrene, PS) is described. In contrast to the, thus far, used two-step procedure ((1) assembly of a PS template bed and (2) careful filling of the voids between the PS spheres), which is restricted to macro structuring, we are able to simply mix the PS template and a commercially available silazane precursor HTT-1800 in toluene. The key is the alteration of the zeta potential of the PS template leading to a homogeneous dispersion in the silazane-toluene mixture. Removal of solvent gives rise to a highly ordered PS-silazane nano-composites and subsequent pyrolysis leads to mesoporous silicon carbonitride (SiCN) materials. The one-pot procedure has two advantages: easy upscaling and the use of PS spheres smaller than 100 nm in diameter, here 60 nm. The PS template was characterized by photon correlation spectroscopy, zeta potential measurements, scanning electron microscopy (SEM), and thermal gravimetric analysis (TGA). The resulting mesoporous SiCN materials were analyzed by SEM, transmission electron microscopy (TEM), nitrogen sorption analysis, and Fourier transform infrared measurements (FT-IR).

## 1. Introduction

Polymer derived (PD) silicon carbonitride (SiCN) ceramics are diversely used materials due to their easy processability, chemical resistance, and high thermal stability [1,2,3,4,5,6,7,8,9,10,11,12,13,14]. Among many applications, the use of PD-SiCN ceramics as a promising catalyst support material (M@SiCN) has been described [15,16,17,18,19,20,21,22]. Especially interesting is the generation of very small late transition metal nano particles from metallo-polysilazanes [17,18,19,20,21,22]. A catalytic reactivity as efficient as for homogeneous catalysts has been observed for Ir@SiCN [22]. Unfortunately, the M@SiCN catalysts developed thus far show a low specific surface area, which means most of the metal nano particles are not accessible. In this context, the generation of nano-structured high surface area PD-SiCN ceramics is a desirable goal. Such structuring is difficult due to the hydrolysis sensitivity of the polymeric precursors. Out of the methods described thus far, the use of polyolefin templates seems most promising [10].

The group of Wiesner [23] used poly(isoprene-*block*-dimethylaminoethylmethacrylate) (PI-*b*-PDMAEMA) as structure-directing agent leading to a meso-structured SiCN ceramics. Furthermore, they combined their synthesis route using PI-*b*-PDMAEMA as structure-directing agent with polystyrene (PS) spheres as templates to structure at various lengths scales [17]. The group of Kim [24] synthesized poly(vinyl)silazane-*block*-polystyrene (PVSZ-*b*-PS) with self-assembly behavior, which was subsequently converted into an ordered mesoporous SiCN ceramic. Moreover, they combined photolithography and advanced nanofabrication processes resulting in a mesoporous SiCN patterns [25]. Jones and Lodge [26] introduced a hard template inverse replication technique. A microphase-separated polymer blend was used for the formation of a PE (polyethylene) template. The subsequent synthesis led to disordered 3D continuous porous non-oxide ceramics with pores between 60 and 100 nm. Our group produced ultrathin SiCN fibers as well as lamellar morphologies performing a one pot self-assembly and organic-inorganic block copolymer synthesis [27]. A commercially available polysilazane acted as the inorganic block and hydroxy-terminated polyethylene synthesized via coordinative chain transfer polymerization [28] as the organic block component.

The groups of Kim and Kenis [15] established the self-sacrificial template method using PS spheres. A packed bed of PS spheres is assembled in the first step and macroporous SiCN (and SiC) monoliths are obtained after infiltrating the template assembly by a preceramic silazane polymer and subsequent pyrolysis [29,30].

The elegant nano-structuring methods applied thus far have certain limitation. The block copolymer based strategies do either introduce oxygen using acrylic monomers or involve sophisticated block copolymer synthesis. Furthermore, bulk material structuring is demanding. The simple PS template approach has been restricted to macro structuring thus far. Most likely, since infiltration into the beds of PS smaller than 100 nm in diameter is challenging. Polysilazane intrusion into such small voids is very slow.

Herein we report on a simple one-pot synthesis of well-defined PS-silazane nano-composites. In contrast to the so far used two-step procedure: first, settling of the PS templates and, second, careful filling of the voids between the PS spheres, we are able to simply mix the PS templates and a commercially available silazane precursor in common organic solvents. The key is the alteration of the zeta potential of the PS template to allow homogeneous dispersion of the PS template in the silazane solvent mixture. Removal of the solvent gives rise to the nano-composites and pyrolysis leads to meso-structured SiCN materials. The one-pot procedure has two advantages: easy upscaling and the use of PS spheres smaller than 100 nm in diameter.

Porous ceramic produced at lower temperatures (900 °C) may be useful for battery applications.

## 2. Results and Discussion

### 2.1. Synthesis of the PS_60_ Template and the PS_60_SiCN Ceramics

**Scheme 1:**
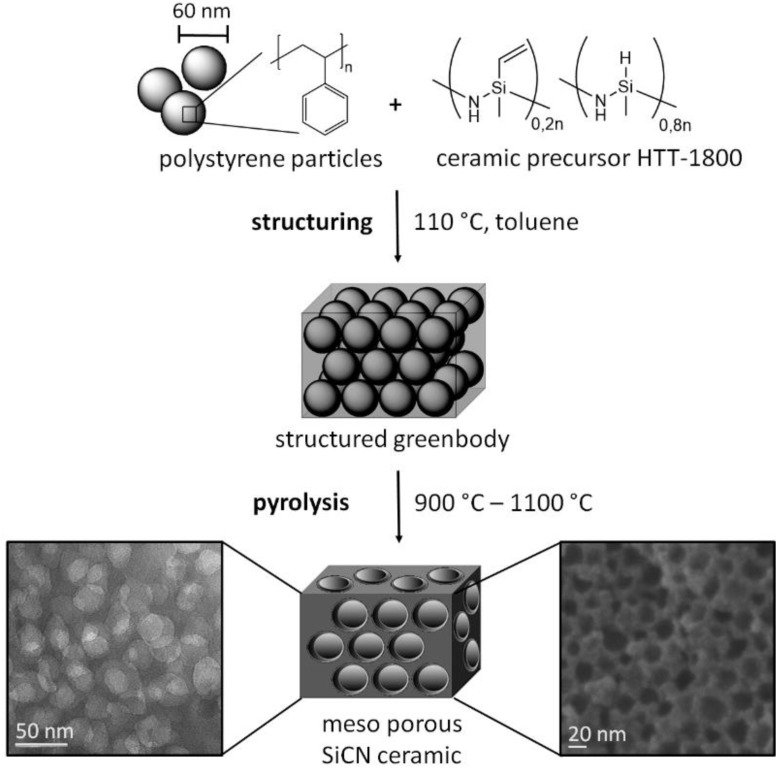
Synthesis route leading to meso-structured SiCN ceramics: (1) Structuring: PS_60_ dispersion in silazane-toluene mixture; pre-crosslinking at 110 °C; removal of solvent, and crosslinking at 110 °C leading to structured green bodies; (2) Pyrolysis with tailored pyrolysis program at 900, 1000, or 1100 °C obtaining PS_60_SiCN_900_, PS_60_SiCN_1000_, and PS_60_SiCN_1100_.

In the first step, the spherical PS particles were synthesized with a diameter of 60 nm (PS_60_) via emulsion polymerization. 4.50 g purified styrene (43.23 mmol) and 0.40 g divinylbenzene (3.07 mmol) were dispersed under stirring in degased ultrapure water. Furthermore, 0.25 g of the surfactant CTAB (0.69 mmol) and 10 mg of the initiator 2,2'-azobis(2-methyl-propionamidine)dihydrochloride (0.04 mmol) were each dissolved in 5 mL ultrapure water. CTAB was added to the dispersion of styrene and divinylbenzene at 80 °C. After 30 min the polymerization was started by adding the initiator and, after 24 h, the polystyrene particles were purified by dialysis and isolated by freeze-drying. The template size of 60 nm is located in the macro scale range and accommodates the shrinking process of the particles to meso size during the pyrolysis [29]. It was essential to generate PS particles with a positive partial charge in order to stabilize a homogeneous dispersion of PS_60_ in toluene during the structuring step. Particles with a negative partial charge, using potassium persulfate as an initiator, dispersed significantly less well in toluene. The commercial available preceramic polymer HTT-1800 was added obtaining a homogeneous suspension of the polymer template and the preceramic polymer by simple mixing. Cross-linking of HTT-1800 was achieved using the radical initiator dicumylperoxide (DCP) at 110 °C. The ordered polystyrene spheres were sealed in the HTT-1800 matrix. Removing the solvent under vacuum led to a structured green body. To guarantee a comprehensive structuring, the mixing ratio of 2:1 of the PS_60_ template and the ceramic precursor is essential. We tested a few ratios based on dense packing of PS spheres and the complete filling of the voids by HTT-1800 (2.5:1 ratio). The best structuring was observed at a 2:1 ratio. Larger amounts of HTT-1800 gave rise to partially non-structured materials. The meso-porous structured ceramics PS_60_SiCN_900_, PS_60_SiCN_1000_, and PS_60_SiCN_1100_ were obtained after the pyrolysis of the green body under nitrogen atmosphere at different temperatures (900–1100 °C) with a tailored pyrolysis program (Scheme 1).

### 2.2. Characterization of the PS_60_ Template

**Figure 1:**
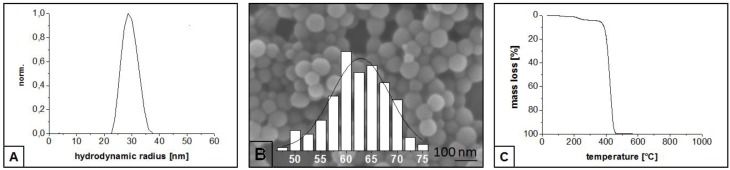
Particle size distribution calculated by contin analysis (**A**); Scanning electron microscopy (SEM) image with particle size distribution (nm) (**B**); and thermal gravimetric analysis (TGA) analysis under nitrogen atmosphere (**C**) of the PS_60_ template.

The hydrodynamic radius of the PS particles was calculated by contin analysis (Figure 1A). A narrow particle distribution in the range from 23.7 to 36 nm was achieved. The peak maxima is at 28.6 nm which means an average diameter of 57.2 nm for the PS particles (PS_60_). Furthermore, a monodisperse behavior is verified.

Particle size and shape of the PS_60_ template were observed by SEM. The particle size distribution was determined based on the scanning electron microscopy (SEM) image (Figure 1B). An average particle size of 62.8 nm was calculated by Gaussian fit. Moreover, a narrow monodisperse distribution of spherical particles was obtained, which is in agreement with the results of the PCS measurement.

By choice of the initiator and the surfactant the PS particles were generated with a positive partial charge, which was confirmed by zeta potential measurements. The PS_60_ template exhibits a zeta potential of 47 mV. Compared to PS particles with negative partial charges, the stabilization of a homogeneous dispersion in nonpolar solvents like toluene is possible enabling the performed one-pot synthesis.

The decomposition temperature of the PS_60_ template was investigated by TGA under nitrogen atmosphere. The major mass loss occurs between 380 and 445 °C. The PS_60_ template is totally decomposed at a temperature of 470 °C (Figure 1C). It was important to investigate the decomposition behavior of the template in order to adjust the pyrolysis temperature (0.5 °C·min^−1^ between 400 and 500 °C). Thus, the entire elimination of the PS template and the generation of maximum pore density was ensured.

### 2.3. Characterization of the PS_60_SiCN Ceramics

**Figure 2:**
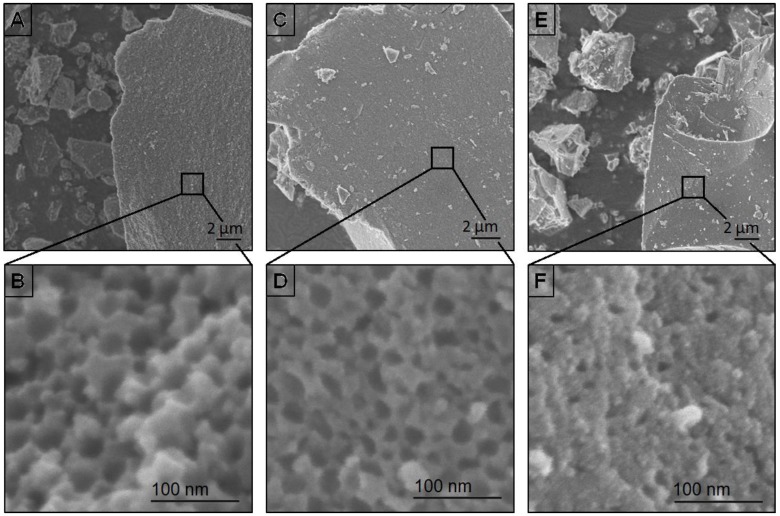
SEM-images of PS_50_SiCN_900_ (**A**,**B**); of PS_50_SiCN_1000_ (**C**,**D**); and of PS_50_SiCN_1100_ (**E**,**F**).

The PS_60_SiCN compounds were pyrolyzed at 900, 1000, and 1100 °C to investigate the temperature dependent stability of the pores. Porous ceramic produced at low temperatures (900 °C) may also be useful for battery applications [31,32]. The SEM-images identify a honeycomb surface structure with small mesopores for the ceramics PS_60_SiCN_900_ (Figure 2A,B) and PS_60_SiCN_1000_ (Figure 2C,D). According to Kim and coworkers [29] a shrinking process of the PS particles takes place during the pyrolysis of the green body. The total collapse of the surface pores is observed at a pyrolysis temperature of 1100 °C (Figure 2E,F).

**Figure 3:**
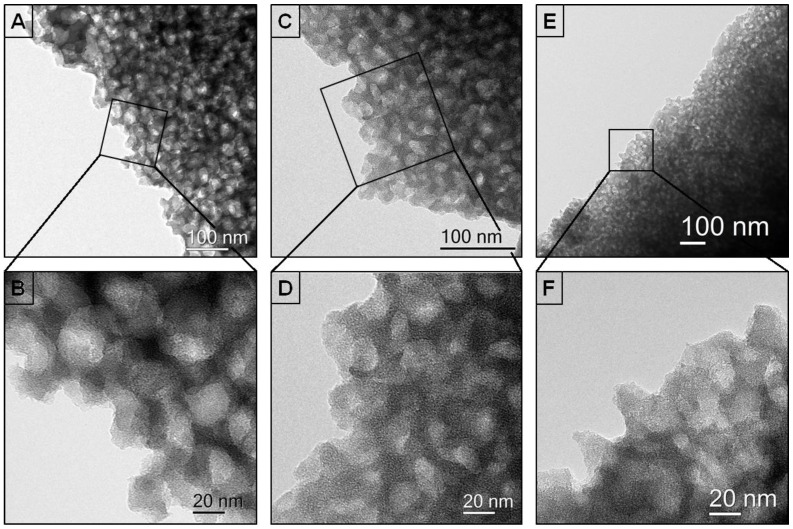
Transmission electron microscopy (TEM)-images of (**A**,**B**); of PS_50_SiCN_1000_ (**C**,**D**); and of PS_50_SiCN_1100_ (**E**,**F**).

TEM-images illustrate the correlation of the pore density and the pyrolysis temperature. Increasing temperatures result in the reduction of the pore density (Figure 3A–F), which confirms the results of the SEM measurements.

**Figure 4:**
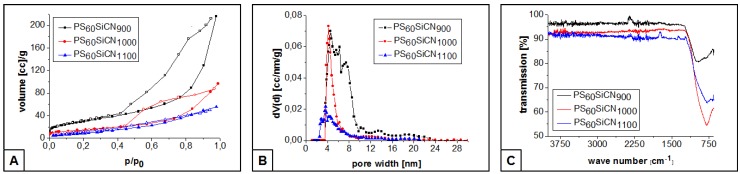
Nitrogen sorption isotherms (**A**); calculated pore size distribution (**B**); and Fourier transform infrared measurements (FT-IR) spectra (**C**) of the ceramics PS_60_SiCN_900_, PS_60_SiCN_1000_, and PS_60_SiCN_1100_.

Nitrogen sorption measurements (Figure 4A) of the ceramics show typical Type IV isotherms according to Sing *et al.* [33]. The presence of mesopores is indicated by the hysteresis. Large specific surface areas (PS_50_SiCN_1100_: 35 m^2^/g, PS_50_SiCN_1000_: 50 m^2^/g and PS_50_SiCN_900_: 110 m^2^/g) correlate with the reduction of pyrolysis temperature indicating the increased mesopore percentage. The calculated pore size distribution (NLDFT) shows a major pore volume between 4 and 10 nm. Furthermore, larger mesopores up to 24 nm can be observed (Figure 4B). The larger pores are attributed to the surface-located cavities according to the SEM-images. The smaller pores are located inside the material. This trend can also be recognized for the ceramics PS_50_SiCN_1000_ and PS_50_SiCN_1100_. With rising pyrolysis temperature the contribution of high range mesopores decreases, which is in agreement with the nitrogen sorption isotherms.

The FT-IR measurements indicate the presence of SiCN ceramics in regard to the characteristic signals of the HTT-1800 precursor (Figure 4C) [34,35]. The broad peak at 1250 cm^−1^ is typical for SiCN ceramics and accrues from the overlapping of the Si-C-, the Si-N- and the Si-N-Si-bands [32,33].

## 3. Experimental Section

### 3.1. Materials and Methods

All reactions were carried out in a dry argon or nitrogen atmosphere using standard Schlenk or glove box techniques. Non-halogenated solvents were dried over sodium benzophenone ketyl and distilled. (1-Hexadecyl)trimethylammonium bromide (CTAB) (98% purity, abcr, Karlsruhe, Germany), 2,2'-azobis(2-methylpropion-amidine)dihydrochloride (97% purity, Aldrich Chemistry, Steinheim, Germany), KiON HTT1800 (Clariant Advanced Materials GmbH, Frankfurt, Germany) and dicumylperoxide (97% purity, Aldrich Chemistry, Steinheim, Germany) were purchased from commercial sources and used without further purification. Styrene (>99% purity, Sigma Aldrich, Steinheim, Germany) and divinylbenzene (technical grade, 55%, Aldrich Chemistry, Steinheim, Germany) were destabilized over an alumina B column (ICN Biomedicals GmbH, Eschwege, Germany).

Photon correlation spectroscopy (PCS) measurements were carried out with an ALV DLS/SLA-SP 5022F laser goniometer system. A Ne/Ar ion laser (λ = 632.8 nm) with a constant output of 260 mW was used as power source. The correlation function was generated by an ALV-5000/E multiple tau digital correlator. The decalin bath temperature was regulated to 20 °C with a computer-operated thermostat. The fixed angle measurements were performed with a 90° angle. The data analysis was accomplished by the CONTIN analysis.

Zeta potential measurements were performed with a Zetasizer Nano ZS (Malvern Instruments Limited, Herrenberg, Germany).

Thermal gravimetric analysis (TGA) were performed under nitrogen atmosphere using a Thermowaage L81 (Linseis, Selb, Germany) and a heating rate of 5 K·min^−1^ up to 900 °C.

CHN analyses were carried out on a Vario elementar EL *III*.

Ceramization was performed in a high temperature furnace (Gero, Berlin, Germany) under nitrogen atmosphere. The pyrolyzed ceramics were milled in a ball mill “Pulverisette 0” (Fritsch, Idar-Oberstein, Germany) for 20 min.

SEM measurements were carried out using a Zeiss Field-Emission-Scanning-Electron-Microscope (FESEM) “LEO 1530 GEMINI”. The acceleration voltage was 1–5 kV. The samples were sputter-coated with a 1.3 nm layer of platinum.

Transmission electron microscopy (TEM) measurements were performed using a Varian LEO 9220 (120 kV, Carl Zeiss, Oberkochen, Germany) instrument. The samples were suspended in chloroform and sonicated for 5 min. Two microliters of the suspension were placed on a CF200-Cu-grid (Electron Microscopy Sciences, Hatfield, PA, USA) and allowed to dry.

FT-IR measurements were performed using a Perkin-Elmer FTIR Spectrum 100 over a range from 4400 to 650 cm^−1^.

Nitrogen sorption analyses were conducted using a Nova2000e (Quantachrome, Odelzhausen, Germany) instrument. The specific surface areas were calculated using *p*/*p*_0_-values from 0.05 to 0.31 (BET). The pore width and average pore volume was calculated by DFT calculations (N_2_ at 77 K on carbon (slit pore, NLDFT equilibrium model)).

### 3.2. Preparation

*Synthesis of the PS_60_ template*: The emulsion polymerization of the cross-linked polystyrene latex particles with 60 nm diameter were carried out in a three neck round bottom flask with a reflux condenser, a KPG stirrer and a septum. 4.50 g purified styrene (43.23 mmol) and 0.40 g divinylbenzene (3.07 mmol) were dispersed under stirring in 90 mL degased ultrapure water. An amount of 0.25 g of the surfactant CTAB (0.69 mmol) and 10 mg of the initiator 2,2'-azobis(2-methyl-propionamidine)dihydrochloride (0.04 mmol) were each solved in 5 mL ultrapure water. CTAB was added to the dispersion at 80 °C under stirring with 200 rpm. After 30 min the polymerisation was started by adding the initiator. After 24 h the polystyrene particles were purified by dialysis and isolated by freeze drying.

*Synthesis of the PS_60_SiCN compounds*: In a round bottom Schenk flask 1.0 g PS_60_ were degassed applying a vacuum of 10^−3^ mbar for several hours to remove residual water. The PS_60_-particles were dispersed in 40 mL toluene under stirring. Subsequently, 0.56 g of KiON HTT1800 (7.77 mmol) and 0.05 g dicumylperoxide (1.85 mmol) were added. Without stirring, the suspension was heated to 110 °C for 20 h. The solvent was removed under vacuum and the *in situ* structured preceramic polymer was annealed for 20 h at 110 °C to complete the crosslinking. The PS_50_SiCN_900-1100_ green bodies were pyrolyzed under nitrogen flow according to the following program:

$$RT\overset{1\;K\;min^{-1},\;3\;h}{\to }\;300\;{}^{\circ}C\overset{1\;K\;min^{-1},\;3\;h}{\to }\;400\;{}^{\circ}C\;\overset{0.5K\;min^{-1},\;3h}{\to }500\;{}^{\circ}C\overset{1\;K\;min^{-1},\;4\;h}{\to }\;600\;{}^{\circ}C\overset{0.5\;K\;min^{-1},\;0\;h}{\to }\;700\;{}^{\circ}C\overset{1\;K\;min^{-1},\;0.5\;h}{\to }\;900\;{}^{\circ}C-1100\;{}^{\circ}C$$

## 4. Conclusions

To the best of our knowledge, meso-porous structured SiCN nano composites were generated by the self-sacrificial template method for the first time. The processability of monoliths was shown in a one pot synthesis including PS latex particles with the size of 60 nm as template and the commercial inexpensive HTT-1800 as preceramic polymer. The positive partial charge of the polymer template facilitates a homogeneous dispersion of PS_60_ particles in the silazane solvent mixture enabling easy upscaling. The influence of different pyrolysis temperatures was investigated regarding the stability of the pores. The specific BET surface area and the mesopore percentage correlates with the decrease of the pyrolysis temperature.

For future research, meso-porous SiCN compounds are well-suited materials for the stabilization of metal particles, which provides the application as catalyst supports.

## References

[1] Riedel R., Kleebe H.-J., Schönfelder H., Aldinger F. (1995). A covalent micro/nano-composite resistant to high-temperature oxidation. Nature.

[2] Weibelzahl W., Motz G., Suttor D., Ziegler G. (1999). Corrosion stability and mechanical properties of polysilazane-derived SiCN-ceramics. Key Eng. Mater..

[3] Kroke E., Li Y.-L., Konetschny C., Lecomte E., Fasel C., Riedel R. (2000). Silazane derived ceramics and related materials. Mater. Sci. Eng. R.

[4] Greil P. (2000). Polymer derived engineering ceramics. Adv. Eng. Mater..

[5] Kleebe H.J., Störmer H., Trassl S., Ziegler G. (2001). Thermal stability of SiCN ceramics studied by spectroscopy and electron microscopy. Appl. Organomet. Chem..

[6] Riedel R., Mera G., Hauser R., Klonczynski A. (2006). Silicon-based polymer-derived ceramics: Synthesis properties and applications—A review. J. Ceram. Soc. Jpn..

[7] Studart A.R., Gonzenbach U.T., Tervoort E., Gauckler L.J. (2006). Processing routes to macroporous ceramics: A review. J. Am. Ceram. Soc..

[8] Colombo P., Mera G., Riedel R., Sorarù G.D. (2010). Polymer-derived ceramics: 40 years of research and innovation in advanced ceramics. J. Am. Ceram. Soc..

[9] Colombo P., Sorarú G.D., Riedel R., Kleebe A., Stech D.E. (2010). Polymer Derived Ceramics.

[10] Shi Y., Wan Y., Zhao D. (2011). Ordered mesoporous non-oxide materials. Chem. Soc. Rev..

[11] Zaheer M., Schmalz T., Motz G., Kempe R. (2012). Polymer derived non-oxide ceramics modified with late transition metals. Chem. Soc. Rev..

[12] Ionescu E., Kleebe H.J., Riedel R. (2012). Silicon-containing polymer-derived ceramic nanocomposites (PDC-NCs): Preparative approaches and properties. Chem. Soc. Rev..

[13] Mera G., Navrotsky A., Sen S., Kleebe H.-J., Riedel R. (2013). Polymer-derived SiCN and SiOC ceramics—Structure and energetics at the nanoscale. J. Mater. Chem. A.

[14] Bernardo E., Fiocco L., Parcianello G., Storti E., Colombo P. (2014). Advanced ceramics from preceramic polymers modified at the nano-scale: A review. Materials.

[15] Sung I.K., Mitchell C.M., Kim D.P., Kenis P.J.A. (2005). Tailored macroporous SiCN and SiC structures for high-temperature fuel reforming. Adv. Funct. Mater..

[16] Mitchell C.M., Kim D.P., Kenis P. (2006). Ceramic microreactors for on-site hydrogen production. J. Catal..

[17] Kamperman M., Burns A., Weissgraeber R., van Vegten N., Warren S.C., Gruner S.M., Baiker A., Wiesner U. (2009). Integrating structure control over multiple length scales in porous high temperature ceramics with functional platinum nanoparticles. Nano Lett..

[18] Glatz G., Schmalz T., Kraus T., Haarmann F., Motz G., Kempe R. (2010). Copper-containing SiCN precursor ceramics (Cu@SiCN) as selective hydrocarbon oxidation catalysts using air as an oxidant. Chem. Eur. J..

[19] Schmalz T., Kraus T., Günthner M., Liebscher C., Glatzel U., Kempe R., Motz G. (2011). Catalytic formation of carbon phases in metal modified, porous polymer derived SiCN ceramics. Carbon.

[20] Zaheer M., Motz G., Kempe R. (2011). The generation of palladium silicide nanoalloy particles in a SiCN matrix and their catalytic applications. J. Mater. Chem..

[21] Zaheer M., Keenan C.D., Hermannsdörfer J., Roessler E., Motz G., Senker J., Kempe R. (2012). Robust microporous monoliths with integrated catalytically active metal sites investigated by hyperpolarized ^129^Xe NMR. Chem. Mater..

[22] Forberg D., Obenauf J., Friedrich M., Hühne S.-M., Mader W., Motz G., Kempe R. (2014). The synthesis of pyrroles via acceptorless dehydrogenative condensation of secondary alcohols and 1,2-amino alcohols mediated by a robust and reusable catalyst based on nanometer-sized iridium particles. Catal. Sci. Technol..

[23] Kamperman M., Garcia C.B., Du P., Ow H., Wiesner U. (2004). Ordered mesoporous ceramics stable up to 1500 degrees C from diblock copolymer mesophases. J. Am. Chem. Soc..

[24] Nghiem Q.D., Kim D.J., Kim D.P. (2007). Synthesis of inorganic–organic diblock copolymers as a precursor of ordered mesoporous SiCN ceramic. Adv. Mater..

[25] Nguyen C.T., Hoang P.H., Perumal J., Kim D.P. (2011). An inorganic-organic diblock copolymer photoresist for direct mesoporous SiCN ceramic patterns via photolithography. Chem. Commun..

[26] Jones B.H., Lodge T.P. (2009). High-temperature nanoporous ceramic monolith prepared from a polymeric bicontinuous microemulsion template. J. Am. Chem. Soc..

[27] Pillai S.K., Kretschmer W.P., Denner C., Motz G., Hund M., Fery A., Trebbin M., Forster S., Kempe R. (2013). SiCN nanofibers with a diameter below 100 nm synthesized via concerted block copolymer formation, microphase separation, and crosslinking. Small.

[28] Pillai S.K., Kretschmer W.P., Trebbin M., Forster S., Kempe R. (2012). Tailored nanostructuring of end-group-functionalized high-density polyethylene synthesized by an efficient catalytic version of Ziegler’s “Aufbaureaktion”. Chem. Eur. J..

[29] Yan J., Hong L.Y., Wang A.J., Kim D.P. (2007). Facile synthesis of SiCN ceramic foam via self-sacrificial template method. Solid State Phenom..

[30] Xiao Z., Wang A., Kim D.-P. (2010). 3D macroporous SiCN ceramic patterns tailored by thermally-induced deformation of template. J. Mater. Chem..

[31] Dibandjo P., Graczyk-Zajac M., Riedel R., Pradeep V.S., Soraru G.D. (2012). Lithium insertion into dense and porous carbon-rich polymer-derived SiOC ceramics. J. Eur. Ceram. Soc..

[32] Song T., Xia J., Lee J.-H., Lee D.H., Kwon M.-S., Choi J.-M., Wu J., Doo S.K., Chang H., Park W.I. (2010). Arrays of sealed silicon nanotubes as anodes for lithium ion batteries. Nano Lett..

[33] Sing K.S.W., Everett D.H., Haul R.A.W., Moscou L., Pierotti R.A., Rouquerol J., Siemieniewska T. (1984). Reporting physisorption data for gas/solid systems. Pure Appl. Chem..

[34] Kriegsmann H., Beyer H. (1961). Spektroskopische untersuchungen an Siliciumverbindungen. XIV. IR- und Ramanspektren einiger substituierter Disilylacetylene. Z. Anorg. Allg. Chem..

[35] Choong Kwet Yive N.S., Corriu R.J.P., Leclercq D., Mutin P.H., Vioux A. (1992). Silicon carbonitride from polymeric precursors: Thermal cross-linking and pyrolysis of oligosilazane model compounds. Chem. Mater..

